# “The hysteria of a little flu”: effects of COVID-19 on HEIs in Brazil

**DOI:** 10.1007/s10734-022-00887-9

**Published:** 2022-07-22

**Authors:** Katucha Bento

**Affiliations:** 1 University of Edinburgh, Edinburgh, UK; 2 Free Afro-Brazilian University (UNAFRO), São Paulo, Brazil

**Keywords:** Public Brazilian HEIs; Colonial legacy, Institutional negligence, Necropolitics, Margin

## Abstract

This essay will look at the key challenges public Higher Education Institutions (HEIs) faced in Brazil during the COVID-19 lockdown. The pandemic led universities to close their campuses and adopt an online interface for academic activities. However, many of these institutions do not have the technological infrastructure for such, nor did the staff and students who suffered further social exclusions. The president of Brazil referred to the pandemic as a “little flu” and later on responded to measures adopting the lockdown as a “hysteric” act that “will lead to an economic crash”. Considering the lack of support from the government and the process of dismantling resources for public HE since the beginning of the new presidency elected in 2018, the COVID-19 lockdown quickly revealed the institutional racism, elitism and ableism evident in this administration’s agenda. The consequence of the agenda is the cuts on research funds and lack of infrastructure to provide online classes, as examples of the severe policies that promote the erasure of marginalised groups. Such policies follow Sylvia Wynter’s “Argument”, revealing a code of symbolic life and death of how human order organises itself through the coloniality of power/being. In order to show how such symbolic code is engraved in the Brazilian educational system, this study explores narratives of staff and students from three universities per region of Brazil to identify how the colonial legacies are correlated with postulates of power in the Brazilian HEIs setting during the pandemic. The paper discusses the challenges experienced while keeping the HE sector active during a pandemic that the government has belittled. The conclusions advocate for organised strategies at the union and social movements level to dismantle the colonial occupation put in place in the foundation of the HEIs and reinforced by the current necropolitical administration.

## Introduction: denial science and life

“In a little while, there won’t even be wood left for the caskets” (Emicida, 2015).

This discussion focuses on how staff, scholars and students from public Higher Education Institutions (HEIs) in Brazil face the lockdown regulations and challenges that the context imposes on them. The narratives of the HEIs community helped weave these experiences with the institutional discourses in the Brazilian political sphere. This paper intends to ask how the code of symbolic life and death was present in the experiences of staff, scholars and students of public HEIs during the pandemic? I will start by dismantling the presidential discourses as part of the preamble of this article to serve as a twofold reflection about the effects of COVID-19 on HEIs in Brazil. For the initial part of the discussion, I am talking about taking off the mantle of administrative intentions of Bolsonaro presidency. Following this section, I will present the methodological elements of data collection, considering the power unbalances within academia in Brazil. In the analysis, the participants are the interlocutors of the dismantle metaphor in my reflection concerning the urgency to demolish procedures and policies that do not support Higher Education as a tool for social justice. The structural exclusion was potentialised during the COVID-19 pandemic, calling out for a strategic decolonial discussion regarding the possible futures in Brazilian HEIs.

In this introduction, I intend to address the first meaning of dismantling by revealing the colonial element in the presidential discourse executing the social injustices revealed by the code of symbolic life and death (Wynter, 2003) according to the human dehumanising orderings of Brazilian society. In this introduction, Emicida’s (2015) soundtrack denounces the suffering caused by the laws made by unjust politicians. I will address the expulsion and destruction of traditional lands or the killing of Indigenous and Black peoples aggravated during COVID-19 (de Oliverira, 2020; APIB, 2020), affecting an important portion of Brazilian institutions like public universities. I challenge such presidential discourses that attempt to define who does not matter and which bodies are disposable in the exercise of what Achille Mbembe (2003, 2019) called necropolitics. Mbembe (2003) points to the material destruction of bodies as part of a colonial legacy that dispossesses humanity from the bodies of those considered unworthy, in a dynamic that is not only situated in the right to kill but all the undermining aspects and rights of being. In the Brazilian context, Lelia Gonzalez and Carlos Hasenbalg denounced the racist “negro’s place” by challenging the misleading discourse of a “racial democracy” when so many social inequalities are happening at the centre of race and class disparities (Gonzalez & Hasenbalg, 1982).

The growth of hunger, unemployment, lack of technological access and precarious living conditions or housing had intersectional effects on the population. When looking at the experiences of HEIs staff, scholars and students, there is a particular way intersectional oppressions are operating, as it reproduces the elitist, racist exclusionary system historically present in the foundation of public universities in Brazil. I invoke the concept of necropolitics (Mbembe, 2003, 2019) to address such a colonial dynamic as an exercise to reveal the code that postulates the “status-ordering” of structural hierarchies (Wynter, 2003). Such ordering is projecting the “Color Line drawn institutionally and discursively between whites/nonwhites” (Wynter, 2003, p. 316), showing that it is explicitly part of the participants’ narratives reflecting in the Brazilian context on the impact of COVID-19 in their experiences within the HE institutions. The HEIs experiences are a snapshot for the discussion about the colonial legacy present in Brazilian institutions addressing the capacity to define who matters and who does not, who is disposable and who is not. As shared by one of the participants (Gabriela, Black, student, South) of this study, the impact of COVID-19 is a healthcare and educational issue in Brazil:

“*The pandemic is costing human resources, not just in the deaths, but people are going to great lengths to meet demands arising from the pandemic along with demands that already existed. 22% of the permanent staff (administrative and teaching) of the HEIs contracted COVID in loco. There have been two deaths of loved ones. Maintaining face-to-face administrative work during a pandemic was a shame. Furthermore, they cut 50% of the positions (administrative and adequate teaching staff), which generated anxiety and frustration amid the pandemic in the worst possible way. These are the actions of a true genocide who calls for the death of his people. A flat-Earth-believer in denial of science*”.

The perception of Bolsonaro’s political discourse situates the present governmental programme dismissing the gravity of the COVID-19 pandemic, which increased the mortality rate since the cases started to be registered in February 2020 (Ministério da Saúde, 2021). In one of the first media appearances since the breakout of the pandemic, in March 2020, Jair Messias Bolsonaro addressed the Brazilian population making remarks saying that 90% of the population would not feel or be affected by the virus, only a tiny proportion of the nation would be the victims of COVID-19 (Planalto, 2020). Within the margin of error, it was not an accidental coincidence for such a precise prediction. In the following year, in June 2021, the number of confirmed cases reached 8.85% of the population (18,742,025 in total) and 505,095 deaths (IBGE, 2020; Ministério da Saúde, 2021).^1^ During the March 2020 discourse, Bolsonaro said: “In my particular case, due to my history as an athlete, in case the virus contaminated me, I wouldn’t need to worry or, at most, would feel stricken with a little flu (…)” (Planalto, 2020: 3:21–3:34). Bolsonaro goes on defending the use of chloroquine for the treatment against COVID-19 as he finalises the discourse asking people to keep going without panic or hysteria (Planalto, 2020). His discourse dismisses the scientific studies, international experiences with the pandemic and the number of deaths that exponentially increased after this pronouncement, hand in hand with the structural racism present in Brazil (Gondim et al., 2020). With more than four different Ministers of Health showing the confusing state actions and promotion of fake news about the pandemic, Brazil’s health policies to prevent the virus from spreading have been called “genocidal”, leading to a parliamentary inquiry since April 2021 (Phillips, 2021). Four other participants pointed out in the questionnaire the term “genocide” to describe the current federal government and its correlation with the systematic negligence HEIs received to face the pandemic. As Mbembe (2019, p. 36) points out:

“Terror and atrocities are justified by the desire to eradicate the corruption of which still existing tyrannies are allegedly guilty. In appearance, terror and atrocities thus form part of an immense therapeutic liturgy, mixing in with which is the desire for sacrifice, messianic eschatologies, the debris of knowledge forms linked either to native imaginations of the occult or modern discourses of utilitarianism, materialism, and consumerism”.

The naturalisation of deaths was part of his political history, which allows me to provide a broader context concerning the necropolitical agenda. An iconic example was the 1999 interview with Bandeirantes TV when he said “a civil war that kills about 30 thousand people” would solve the countries problems like the military regime did not manage to do (BN, 1999; Globo, 2019). His discourse about the need of having an armed society (Casado & Londoño, 2020; Fernandes, 2018), stopping the demarcation of Indigenous territories (Resende, 2018), dehumanising speeches comparing Black people with cattle, among other attacks against women, LGBTQ + identities (Carta Capital, 2017) are a few examples of how his presidential campaign was designed. During his administration, there has been an exponential increase of families affected in the rural communities due to violent invasions, according to the report developed by the Pastoral Land Commission (CPT, 2020). Between 2018 and 2020, the number of families affected increased from 14,757 to 81,225, representing mainly Indigenous communities, followed by quilombolas (CPT, 2020). There was a 295% rise in 2 years related to land violence (invasion or falsification of ownership using force). Indigenous communities are also more affected by the killings in the rural contexts, representing 39% of assassinations (CPT, 2020) usually silenced or not captured in the media radar. Necropolitics is what Mbembe (2003, 2019) called a system that massacres, displaces, confines or interns in camps.

The expulsion of Indigenous and Black communities from their historical territories situates institutional execution of social injustice with genocidal consequences (Karol & Silva, 2020). The place where people live defines where they will go to study. Marginalised areas—due to displacement or confinement in vulnerable areas—with a majority of Blacks and Indigenous peoples suffer from a lack of formal education and higher surveillance by the police (de Albuquerque & Leandro Ribeiro, 2020). In the national context, assassinations due to violence (especially in encounters with the police) kill 37.8 of Black people compared to 13.9% of non-Blacks (IPEA—Instituto de Pesquisa Econômica Aplicada, 2020). The use of the word “genocide” is carefully used to indicate the systematic killing of Black and Indigenous people, especially by the police (Anistia Internacional, 2017; Anistia Internacional Brasil, 2014; Câmara dos Deputados, 2015a, 2015b; CPT, 2020; Fórum Brasileiro de Segurança Pública, 2017; Fórum Brasileiro de Segurança Pública, 2017; Nascimento, 2016). Eviction-genocide of racialised as nonwhite peoples and native to their lands (in)formed the basis of a dehumanising process that remains at the roots of capitalism and modernity (Quijano, 2000). The eviction-genocide in Brazil is essential to be mentioned as evidence of the colonial dynamic that root intuitions and the social fabric erupting in the context. The data mentioned also points to the intersectional element of racism, in which gender, class and sexuality show how necropolitics is constituted in land dispossession, hunger, lack of health and educational access as historically present in Brazil and increasing during the pandemic.

The destruction of nonwhite bodies and populations in the Brazilian context reveals how necropolitics (Mbembe, 2019) is present in the institutional destruction and disposal of human bodies. The link with such violence is present in the data of COVID-19 deaths is that the racial inequality follows the disproportional mortality rates in racialised Indigenous and Black populations. The Brazilian population is composed of 56.2% of Blacks, 42.7% whites and 1.1% of Indigenous and “Yellow” peoples (PNAD, 2019). As Gondim et al. (2020) presented in their study, the “COVID-19 does not reach groups and places symmetrically and democratically; it is in the marginalised areas and favelas, places with the worst indicators of human development, that the pandemic presents its cruellest face”. The Black and Indigenous areas are more affected by the COVID-19 (Gondim et al., 2020). Despite having a public healthcare system (SUS—*Sistema Único de Saúde*), the hospitals are concentrated in central areas and middle-class urban regions where white people predominantly live (de Albuquerque & Leandro Ribeiro, 2020; NEXO, 2020; Souza, 2017; Werneck, 2016). This data reflects on the historiography of racialisation politics in Brazil from the intersectional harm that is forged by the myth of a racial paradise in the social imaginary. The grounds of the Brazilian society as the slave trade and land exploitation is portrayed as the reason for peaceful coexistence between the three races of Indigenous, Black and white forming the nation and a class of “free men” making up the idea of a Brazilian racial democracy, as if economic and social opportunities would be equal to all (for more about racialisation politics in Brazil see Bento, 2017; Gomes, 2005; Gonzalez & Hasenbalg, 1982; Guimarães, 2010; Paixão & Carvano, 2008).

Necropolitics in Brazil navigates the governmental institutions as part of the elements that erected values and practices of such spaces. It arrives at the public HEIs with the historical, social exclusion maintained by colonial influences to this day. In the following sections, I will present the colonial making of the public HEIs in Brazil and the methodological challenge to this study. The design of data collection/generation and analysis of a project meant to collect narratives of three public HEIs of each region in Brazil. However, it ended in gathering data of over 69 public HEIs. After this section, such experiences will enable a deeper reflection of what to expect for Brazilian public HEIs in how necropolitics is manifested in institutions that are part of the social inequality in the country from the foundation. I will address the objectification of labour and commodification of education, manifested in narratives such as “*we are treated like numbers as if we don’t have family, personal problems, loved ones. As if deaths and risks were trivial or acceptable*” (Claudemir, white, lecturer, Southeast). Narratives of experiences during COVID-19 design the following topics concerning the near future and think about collective strategies of solidarity that should anticipate any potential utopic abolitionist project.

## Methodological notes

As mentioned above, the initial goal of this research was to gather a small number of narratives to understand the different experiences in three public universities of each region of Brazil: North, Northeast, Midwest, Southeast and South. The research organised a clipping about the public documents issued by the Ministry of Education and the HEIs about the pandemic to analyse institutional discourses. For this article, this data will not be fully explored as I aim to centre the experiences of academic staff, scholars and students. Thinking about bringing the decolonial and Black feminist approach to this study, the team of five women (all Black and Brazilian), including me as the leading researcher, organised a multidisciplinary strategy for the data collection/generation. It was relevant to consider the historical, social exclusion and erasure of Northern HEIs, epistemes and voices in Brazil from the starting point.

The geographical, social exclusion is systematically related to the concentration of Black and Indigenous peoples in the Northern and central areas of the country (Souza, 2017). As Jessé de Souza (2017) suggests, the consolidation of Southern regions as the economic, cultural and social references of the dominant—white—elite parts from the slavery inheritance as one of the socio-cultural mechanisms that form social classes (Souza, 2017). Consequently, these Northern and Midwestern regions are overlooked at the drought areas in need of irrigation and water access. The federal government facilitated the violent expulsion of traditional communities from their lands, among other atrocities that made the scenario of hunger, unemployment and violence the country’s necropolitical landscape. In this paper, I present how Northern and Midwestern regions remain institutionally ostracised as important epicentre of knowledge production in research and education (Moreira, 2021).

We asked ourselves: How can we organise small-scale research and avoid reproducing the hegemonic narrative that uses positivist methodologies to justify the predominant presence of Southern (Southeast and South) voices, references and realities in the discussion? In order to counter such a colonial erasure, the rationale of this methodology is to centre the margin (hooks, 1984) to understand the multiple experiences that are present in Higher Education Institutions during the COVID-19 pandemic, especially in the systematically overlooked regions. By starting the data collection/production in the Northern regions of Brazil, we managed to reach a similar proportion of participants in Brazil instead of having participants proportionally represented by the number of universities in each region. The social inequality present in Brazil calls attention to the unequal distribution of free Higher Education access in the country as part of the coloniality of power (Quijano, 2000).

This study covers a small universe of HEIs in Brazil that suggests a different approach in the discussion. In the 2018 Census of HEIs in Brazil, 63.2% of the 299 public HEIs in the country are situated in the South and South-Eastern regions (INEP, 2018). Using this statistical information, the strategy was to turn the map “upsidedown”, aiming the initial recruitment at Northern and Central regions as a “centring the margin” (hooks, 1984) strategy. In total, 53.8% were from the North, North-East and Midwest regions, intending to illustrate the narratives and experiences of people working and studying in Brazilian public HEIs. There is no intention to draw comparisons and represent the Brazilian society or universities but dis-mantle discourses that remain evoking codes of life and death determined by the coloniality of power by framing a counter-narrative with people (in this case, participants of this study) living the outcomes of institutional decisions during the pandemic.

The study was 16 months-long, divided into five actions: (1) design of an online questionnaire; participant recruitment using the centring the margin (hooks, 1984) method; (2) collection of responses; (3) develop a clipping of discourses and reports from the federal government and the announcements and administrative documents published by the HEIs since February 2020 about the procedures during the pandemic; (4) data treatment—anonymisation, graphs and thematic analysis from the participants’ narratives; and (5) data selection for publications. For the purpose of this paper, the main focus of the discussion will be the results of the online questionnaire, having the information from the clipping as supporting information to amplify the understanding of the impact of COVID-19.

The ethics involving sensitive topics related to the impact of COVID-19 in people’s lives was considered. It was known, and after confirmed by a few narratives, that somehow our participants were mourning or knew of someone in such a situation due to COVID-19 or its complications. In this sense, it was decided to keep answers as open and optional as possible. Open questions also included race and gender identification; witnessed, heard or lived experiences of discrimination; perceptions about HEIs and government responses to COVID-19; and different opinions about the pandemic within the public higher institutional context. With the purpose to promote a conversation regarding the experiences of scholars, staff and students, the main challenge of this methodology was to gather information from all the five regions of Brazil during a short period of 8 months of research and a low budget.

As a result of the online questionnaire disseminated during the first trimester of 2021, 66 responses were collected. The quantitative element that helps to address the founding colonial roots of HEIs in Brazil is the racial self-identification of the participants. The open question resulted in 63% of participants self-identifying as white, pointing to the predominant presence of white people in institutions of prestige and privilege in Brazilian society. The overall order in which access to education as a student or part of the labour market is rigorously conserved by the social hierarchies established in coloniality. The study’s population is evidence of a “‘learning system’ and order of knowledge as articulated in the institutional structure of each order—this was to be no less the case with respect to the projected ‘space of Otherness’ of the Color Line” (Wynter, 2003, p. 316). It is not the intention of this paper to draw regional comparisons from the data, but to highlight that the inequalities in Brazil are not homogeneous, as the spatiality of Otherness is presented through race, region, class and gender.

During the data collection/production process, official documents published by the HEIs about the institutional response to the COVID-19 pandemic were collected on the HEIs websites. The autonomy of these institutions is not the same, depending on independent funding, state administration and federal financial distribution. The latter has been declined or cut since the start of Bolsonaro’s administration in 2019, as Gabriela pointed: “*The federal government cut a lot of funding, which significantly reduced the number of scholarships and grants that could be released when students, faculty and administrative staff needed the most*” (Gabriela, Black, student, South).

As no personal information was requested in the questionnaires, the intention to guarantee anonymity also requested that some information be altered. Part of the collection/production of the study was the gathering of the president of the republic public discourses to weave into this research the background of political scenario that reflected on the decision-making process of the HEIs and livelihood of staff, scholars and students involved in the context. Based on the responses of the participants, topics were raised as pressing issues discussing the effects of the COVID-19 on the public HEIs in Brazil. Codes of life and death are discursively presented in written Portuguese language format. I aimed to translate the English language and the intensity and affective tones shared in the questionnaire and presented in the following discussion.

### Institutional misleading speeches, confusing practices

“*Jair Bolsonaro’s Brazil is a sounding board of hate and horror*”. (Alberto, white, lecturer, South)

The pandemic offered good opportunities to produce and distribute fake news, especially by the president of Brazil. The discourses against the lockdown were justified by the idea to keep the economy alive at the same time when mass graves started being used to bury people dying from COVID-19. As Alberto noted above, the lack of national coordination to prevent, control and treat the COVID-19 dissemination had a vital consequence that reached all HEIs in Brazil. The different regions, states and regions adopted different lockdown criteria and institutional activities (NEXO, 2020). In the words of Carlos Lula, the president of the National Council of Health Secretaries Brazil, “Brazil became a study case of what not to be (…)” (Nexo, 2020). Some states did not need to have closed entirely at once, and other regions released regulations of lockdown with no epidemiological criteria for such a decision (Nexo, 2020). In some universities, the resolutions about the COVID-19 procedures were not homogeneous.

For the federal universities, for example, the Ministry of Education (MEC) published that from 15/12/2019 until 30/06/2020, there were 25 federal institutions of undergraduate programmes with remote activities and three in suspension (MEC, 2020). From 01/07/2020 until 30/06/2021, there are 31 federal HEIs in remote activities, four in hybrid modality and three to be defined. The discrepancy of numbers in total is due to the adherence of HEIs in providing data; some institutions did not have the resources to develop activities and report back to MEC. A participant (Gilberto, white, lecturer, Southeast) said that “*the federal government simply turned its back on the institution and reduced research resources. It was disastrous*”.

Bolsonaro chose to take an anti-vaccination positionality, sharing misleading information about the importance of science such as: “In Pfizer’s contract… It’s there: we [Pfizer] are not held responsible. If you become a chi…, if I become an alligator, if you become a superman, if it starts growing a beard in a woman, or a man starts talking with soft voice… and what’s worse: messing with people’s immune systems” (G1, 2020). Here Bolsonaro is referring to the DNA modification fake news as he makes racist, sexist, transphobic remarks, suggesting that the vaccine would dehumanise people as they would turn into animals (racist remark, especially when he interrupts himself as he was about to say “chimpanzees”) or change their gender identities. This is what Sylvia Wynter (2003, p. 288) calls “formulation of a general order of existence”, as Bolsonaro jokingly traces a line of humanity and validation of truth in his discourse.

Denouncing Bolsonaro reckless comments caused severe consequences in the maintenance of research grants in Brazilian public HEIs in all areas of knowledge. When asked about the federal support for HEIs, participants nearly unanimously responded that there was a lack of institutional response, guidelines and support for HEIs. As an answer, the words “genocide”, “chaos”, “terrible” and “awful” were used many times to assess the federal government response to the pandemic. Many participants denounced the president’s misconduct to legitimise chaos and social exclusion:

“*In our case, the* [response from the federal government was the] *worst possible. In addition to the lack of support in general, budget cuts and defamatory campaigns, there was also the delegitimisation of our consultation process in the dean elections and a difficult period of uncertainty regarding the nomination of the elected slate and intervention*”. (Sofia, white, lecturer, Southeast).

[The federal support was] “*Null. I believe that the deliberations of the academic community found viable answers for the institution’s functioning*”. (Célio, Black, admin staff and student, Northeast)

“*They* [the government] *were terrible. MEC (Ministry of Education) did absolutely nothing apart from using nonsense and lies to accuse professors. They were irresponsible and criminals*”. (Alberto, white, lecturer, South)

“*Attitudes such as cuts in undergraduate and graduate scholarships, cut discretionary funds, and political persecution were the federal government’s disastrous actions*”. (Carlos, Black, lecturer, Midwest)

[About government response] “*Was there action? I don’t even know who the minister of education is anymore; I’ll have to look it up on google. The worst federal government in the country’s history*”. (Patricia, white, student, North)

The experience of scholars, staff and students in public HEIs in Brazil shows a distrust in the federal government. As pointed by Gilberto and Carlos, some participants, such as Patricia, used irony to indicate negligence and the disastrous consequence of discourses against science resulting in cuts for research and students. The confusion generated by the lack of support in HEIs practices and research harmed the democratic process at the faculty level for decision-making and new administrative elections, as presented by Sofia and Célio. The misleading speeches from the federal government questioning whether the pandemic was real and the importance of science is analogue to the systemic pattern of social inequality intensified during the COVID-19 pandemic. The public HEIs depend on public funds and decisions to exist, experienced the consequence of such a pattern that causes profound negative impacts in the infrastructure of how Higher Education is offered for free in the country.

The government neglected internal information produced by the public institutions that could support the effective distribution of resources and address to particular issues of each region. The universities formed their own research observatories, the government produced data about the impact of COVID-19, especially the Ministry of Education and public research institutes to understand how to minimise or avoid negative impacts during the pandemic. Reports such as regular government bulletins, reports on the growth of inequality and unemployment in Brazil during the pandemic, measures for educational access required during the pandemic, including the limitations to reach marginalised regions were assessed, analysed and published by Brazilian public organs (BRASIL, 2020; IPEA—Instituto de Pesquisa Econômica Aplicada, 2020; MEC, 2020). Therefore, the practices that generated confusion and neglection during the pandemic in Brazil were deliberate, further increasing the basic access and security to food, education and public health, which ultimately is shown in the aforementioned intersectional inequality and numbers of deaths.

As mentioned above, public HEIs are elitist spaces with a predominance of the white, middle class and westernised presence. To get to the point of delegitimising these spaces, it means that the continuation of HEIs activities is at the expense of those at the bottom of the social hierarchy and the limited autonomy at the departmental levels at the HEIs. In the next section, I explore how the HEIs can be at the service of a globally hegemonic system of social rules from westernised perspectives that reinforce human-occupied hierarchies into a colonised relation where nonwhite peoples are subjugated (Wynter, 2003). I am translating her Argument (Wynter, 2003) into the design of Brazilian institutions as formal spaces forged by colonial references that reproduce such global hegemonic legacy.

### HEIs responses: effect of exclusions

“*MEC (Ministry of Education) sent a letter tonight saying that we have to report by tomorrow about what we’ve been doing about COVID-19. Since I can’t write that we are crying and cursing the government, I am writing about the research activities*”. (Jaqueline, Black, lecturer and administrative staff, Southeast)

Considering the myriad issues public HEIs in Brazil had to manage due to the federal government’s negligence, it is relevant to recognise the effort at small scales such as departments, local union collectives or research centres to continue academic activities. As Jaqueline pointed above, the attention received by the Ministry of Education (MEC) requesting a short notice report represented an unrealistic demand, especially if compared with the negligence of the same federal government. Jaqueline, among other participants, also pointed that there were positive outcomes.

However, the complexity of speaking about Brazil’s public Higher Education system concerns the roots of a disciplinary paradigm for the organisation and validation of knowledge and truth, serving as an overall system to preserve the status quo. As Sylvia Wynter (2003) presents in her Argument, the status quo refers to the colonial educational system created to negate the humanity of nonwhites, “blocking out” the counter-narratives and knowledge of these groups. In that sense, as much as there were positive results at the cost of the staff and scholars overloaded labour, the institution as a whole reproduced the exclusions that had been increased during the pandemic.

“*Even if they theoretically opposed the president’s denial of the pandemic, the HEIs continued to operate their exclusionary racial policies. (…)One of the main difficulties raised by Black students was to continue their studies doing distance learning. Lack of adequate equipment, no internet access (especially for rural residents), lack of good physical space for studies. The university did not pay due attention to this scenario and continued its activities without these students. In particular, [my university and the funding institution] was putting pressure on my colleagues and me to report what we were doing in our research, threatening that the grant would be cut if we didn’t report. If it wasn’t for the organised student activities, we would not have funding for our dissertations and theses*”. (Paulo, Black, student, Northeast)

Paulo and Jaqueline mention the practice of reporting back as a controlling mechanism of liberal institutions, developed in favour of legislative measures to control and restrict basic rights to nonwhite peoples in the Brazilian context (Morais, 2015). The issue of race is at the core of the colonial question (Wynter, 2003) when addressing the cascading social exclusions that are represented in this article by Bolsonaro’s speeches, the authorisation of perpetuating necropolitics at the institutional levels in Brazil, taking as an example the public HEIs during the pandemic. What Paulo cites above illustrates how racism is the driver of the necropolitical principle that inflicts harm and humiliation under the justification of keeping the order of the “juridicobureaucratic and institutional micro-and macro-measures of the state machine” (Mbembe, 2019, p. 59). Mbembe (2019) cites Brazil as one of the countries where “nanoracism” is part of the obligatory hydraulic racism that is part of institutional discrimination and segregation in the name of a neutral secular democratic state.

“*Exclusion of students without access to equipment and internet that had their access to university activities limited. My supervisee had to finish her report on her mobile phone, as she depended on the university’s computer*”. (Adriana, white, lecturer, Northeast)

Terrorising students to send research reports when many in a situation of exclusion did not have technological access is to turn a blind eye to the intersectional exclusions of class, race and geography that impact Black and Indigenous students living in a situation of social vulnerability and marginalised areas. Paulo and Adriana are situating the technological exclusion after the universities autonomously decided to shut down, countering the president’s discourses. For those students who only had technological access in the universities, it was not a solution to carry out online fieldwork for their research. Technological issues were taken into consideration by the HEIs. However, threatening to cut funds was also part of the domino effect from the federal government’s cuts. As much as it is a complex situation, a clear policy to address systematic exclusions were not implemented with the same consistency as the colonial practices in the Brazilian public HEIs, where again the habitual loss of historically excluded was naturalised.

The outcome is complex, as the universities attempted to develop programmes of technological assessment for students while the federal government trivialised the actions for the pandemic. Bolsonaro was against the closure of schools and universities, leaving to the Ministry of Education (MEC) a loose role in how it would decree measures to educational institutions, including HEIs (MEC, 2020). When analysing the scholarship measures, distributions of technological materials and funds sent to the universities released in March 2020 (MEC, 2020), it does not provide clear criteria to prioritise vulnerable groups. The lack of clarity in terms of support during the pandemic reinforced the intersectional marginalisation in regard to HEIs access, for example. In the overall lack of access to Higher Education, Northern regions had less domestic access to internet through Wi-Fi or mobile access from 3G or 4G. Students from the North had a greater proportion of exclusion representing 2% of undergraduates with no internet access, in comparison to 1.1% in the Northeast; 0.6% in Midwest, 0.4% in Southeast and 0.2% in the South (Castioni et al., 2021).

Lacking guidelines and social policy to address marginalised groups during the pandemic created new effects of exclusion. Digital exclusion is one of the effects of the pandemic in Brazilian HEIs, as illustrated from the cases that follow. A case with students with trans and intersex identities had their gadgets delivered with delay due to an administrative confusion about their social names. Jonas, white, student, from the Northeast, described that receiving a tablet from the government was the only possible way to continue studying. However, the process took over 9 months (one academic year) because the administration did not process their social name, as requested in the enrolment of the course. He said:

“*The system is Transphobic. It should be simple to find me from my social name. The person who took care of the process knows me!*”

The social exclusion in society aggravated during the pandemic, as it remains the case of non-conforming gender identities. The participant shared that the department that processed the gadget was the same staff who managed the requirements to change their social name in the university’s documents. The practices of exclusion aggravated during the pandemic, as students like Jonas were deprived of continuing studying for mistakes, confusions, miscommunication practices situated in discrimination and exclusion of vulnerable groups.

“*Two people [with disability] in the Biology course need particular attention. Even before the Covid-19 pandemic, I noticed a lack of skills of fellow professors in dealing with deaf-mute and an autistic spectrum disorder. I think that got worse*”. (Fabio, Black, lecturer, Northeast)

Ableism was another reported issue by the participants, as there was no specific training about inclusive classrooms to staff and lecturers prior to the pandemic, as Fabio pointed. The word “catastrophic” was used several times by the participants to describe the results of institutional strategy to manage the pandemic. In Viji Kuppan’s (2018) account for racist, ableist, sexist intersecting oppressions from slavery to Trumpism, it is possible to bridge a correlation with Bolsonaro’s administration invested in structures to have trans, queer and disabled people of colour (PoC) occupying the margin of the margin. HE students living in remote areas, areas with non or little phone or internet coverage, communities with restricted technological resources, including those from dissident identities, were the living evidence of the social exclusion that was put in place since the design of such institutions.

My point here is to recognise the efforts of the public HEIs in promoting a counter-narrative to the necropolitics represented by Bolsonaro’s discourses while revealing the underlying colonial structure that maintains the existence of public institutions in Brazil. During the research, the news showed the rise of hunger cases in Brazil, reaching 19.1 million people (RBA, 2021) and 14.8 million people unemployed (Globo G1, 2021b), while participants pointed to the effect of these issues in the classroom, as Vera reveals:

“*I saw, and I heard, many colleagues describe situations of vulnerability and difficulties in self-sustaining. Hunger, unemployment, psychological disorders… and this destabilise me profoundly. I was able to help some of them, and I asked teachers and close friends to help some others*”. (Vera, student, white, Northeast)

As mentioned above, as much as the inequalities could be reproduced internally in the HEIs, the social inequalities also affected the experiences in the HEIs. Hunger intersected with university drop outs, as some students saw themselves in situations between going to class or eating (BBC, 2021). Bambra et al. (2021) book “*The unequal pandemic: COVID-19 and health inequalities*” mentions four predicaments on how the pandemic is experienced. They are as follows: the pandemic kills unequally, the pandemic is experienced unequally, the pandemic impoverishes unequally and the pandemic inequalities are political (Bambra et al., 2021) . These contextualised social exclusions presented effectively relate to the colonial code of symbolic life and death (Wynter, 2003). The unequal element of the colonial code was growing before the pandemic, when Brazil had an overall mark of unemployment and informal precarious jobs of 40% while the Northeastern region had 53% (Ribas, 2020). During the pandemic, the Northeastern state of Bahia reached 20%, the highest unemployment rate in the country (Ribas, 2020), accentuating the pre-existing inequality that Vera shared as part of the experience she shared with close friends and colleagues. Following this reflection, Inês and Rebeca bring other nuanced examples about experiences in the HEIs during the pandemic, not with such direct effect of the pandemic:

*I understand they are doing their best. But as I said, I know some people who have material needs. I also see the news about indigenous people finding it hard to get the vaccine, even though they are a priority group.* (Inês, white, student, Southeast)

*I don’t know them, but I heard that some indigenous people have many difficulties accessing online classes, getting food, supporting themselves, or returning to their communities.* (Rebeca, white, student, Southeast)

Several of our participants equated the social and geographical disparities to access education during the pandemic with higher risks to access basic rights: food, transportation and health care. The domino effect of these inequalities is more than what Bambra et al. (2021) suggest in terms of political inequality, but they are necropolitical (Mbembe, 2019). It is indeed that “the original inequalities leading to these unequal impacts were a result of prior political choices, and policymakers” (Bambra et al., 2021, p. xiv). Such institutional decision-making successfully defines the human archipelago of Otherness, “to be peopled by a new category, one now comprised of the jobless, the homeless, the poor, the systemically made jobless and criminalised” (Wynter, 2003, p. 321). In the Brazilian public HEIs, such archipelago isolates those students with dissident identities bearing the brunt of the impact of the colonial line dividing who lives and who dies during the COVID-19 pandemic. The political organisation of a neoliberal setting is to portray all the issues and inequalities as a personal matter instead of a fundamental constitution of inequality at the public institutional level, as Silvio Almeida says (Betim, 2020). Following this reflection, I take the effect of exclusions to explore the strategies to overcome the negative impacts of the necropolitics in Brazil and the possibilities to think about a different future of public free education.

### Boundaries of colonial occupation and possibilities to challenge necropolitical structures

*We are treated like numbers as if we don’t have family, personal problems, loved ones. As if the deaths and risks were trivial or acceptable. This “new normal” situation is absurd. I haven’t left the house for almost a year, with very few exceptions. I haven’t seen my wife since February 2020 (she’s in Portugal, also in lockdown). I go to sleep and wake up worried about her. I don’t care about the doctorate, the articles “in the oven”, with Lattes* [an online platform for academic curriculum]*, if my family, friends, students and other people are not doing well. What is the point of reaching personal goals and knowing that we have normalised 30,000 deaths/month (not including underreporting)? I feel privileged to have a house, food, job, salary. However, I have kept myself at home to avoid risking other people. I moved to my mother’s house so she wouldn’t be alone (she’s from the risk group), and I don’t consider it safe to “go back* [from lockdown] *little by little” without health support, without mass vaccination, without reduction in transmissions. Sanitation measures are precarious and insufficient, but management bodies speak as if they were totally safe. But we even ran out of water and toilet in the universities, ran out of toilet papers. People’s lives are the most important. (…) It is not “just a little flu”. It’s genocide. So, higher education cannot reproduce this insensitive market logic since it is about human lives. Are we supposed to fulfil the goals of the IES while the students agonise???* (Claudemir, white, lecturer, Southeast)

The diverse experiences while working and studying in a Brazilian public HEIs found similarity in pointing the federal government performance as a barrier to developing Higher Education programmes, research and publications. Claudemir shared in the above narrative a deep reflection about the conditions to continue with the academic programme when so many atrocities are happening. Lurdes also noted the main challenge as a lecturer was:

*[To] teach classes while experiencing my grieving process.* (Lurdes, Black, lecturer, North)

Claudemir and Lurdes bring to light Mbembe’s (2003) “colonial occupation” in the Brazilian public HEIs. The “colonial occupation” (Mbembe, 2003, p. 25) takes over the matter, with power to seize, delimitate and assert control over a physical geographical area, such as the HEIs, setting up social and spatial relations (de Albuquerque & Leandro Ribeiro, 2020). The necropolitical discourses in the public social sphere affected the internal practices in the HEIs, informing the relational bound between the macro and micro productions of hierarchies and code of symbolic life and death (de Albuquerque & Leandro Ribeiro, 2020; Karol & Silva, 2020). Such was the case of how intense Bolsonaro’s administration reproduced severe oppression and poverty based on race, class, gender and geography with peculiar impact on the space of public HEIs in Brazil. The colonial occupation divided the HEIs space into compartments as a way to disperse coherent and unified operations to tackle the pandemic. Bolsonaro’s administration created discuses about science and universities in Brazil to situate HEIs as a place of “ill fame”, isolating HEIs to little infrastructural support to attend to the demands on their own, as Amanda points:

*I see that public HEIs had a good performance, suspending classes and seeking to implement emergency remote teaching in the best possible way. As it is a reality very different from the usual, it still had many challenges, but I see a great effort to deal with problems.* (Amanda, white, student, North)

Participants described that the survival of the public HEIs in Brazil was due to compartmentalised decision-making at college or faculty levels. Amanda recognises the successful results for the urgency of the context. When asked about the emotional and psychological labour in their workload, staff and scholars answered similarly, pointing that “increased a little” and “increased a lot”, only two (white, male, from Southern regions) respondents said that it remained the same. Similar results appeared when asked about the dedicated time represented in the workload during the pandemic. Staff and scholars indicated an intense increase of hours and attention required. These results speak to Amanda’s experience regarding the positive outcome of having professionals in HEIs experiencing an overload of work to continue with the activities. Generally, as Amanda said, students did share the opinion in recognising the local level of HEIs dealing with the pandemic. One of the students noted:

“*My university was quick in responding to the pandemic (…) even though I understand that teachers and administrative workers’ psychological and physical health is costly*”. (Gabriela, Black, student, South)

This case allows exploring how the academic and professional staff were also overwhelmed by the confusing practices adopted during the pandemic. As there was no official federal coordination, the academic and professional staff developed the new practices as “they were playing by the ear”, as the expectations and planning were difficult to situate as the mortality rate increased. Following, Maísa shares her experience from a Southern HEI about how was the interaction with work in the first months after the interruption of classes:

*At the first moment, the classes were totally suspended. Departmental activities continued. We had long departmental meetings, NDE and internship commissions. In the beginning, it was challenging because many colleagues think that you are always available because you are at home. I had a department meeting that lasted 6 hours. The number of sessions and the lack of an established limit made it very complicated to accommodate a research routine, family and home. I saw a meme that hit home. It said: “I’m afraid to open the fridge door and find one more link for a meeting”. Some people say that a professor’s life in this neoliberal world is challenging. Setting time limits for work and self-care, time for the classroom and time for research. But in the pandemic, everything got worse. (…) I consider that I created limits in my approach towards other people, but my colleagues not so much: there were messages at the weekend or messages asking for things at 11 pm. Over the months, we have been adjusting and imposing limits.* (Maísa, white, lecturer, Southeast)

The autonomy reflected on positive experiences and represented a higher responsibility to the HEIs (Castioni et al., 2021). The lack of boundaries in the workload required more attention at the private level since the institutional responses had confusion or unclear guidelines regarding the administrative and pedagogical procedures during the pandemic. Several participants noted having concerns with mental health issues, leaving to the collegial relationships to have empathy and discussions because there was no measure taken place at the departmental or university levels:

“*We had many meetings and tried to make decisions as collective as possible in the School of Education. Some colleagues suspended their activities for some time (1 to 2 weeks) until the classes’ ways of functioning were redefined*”. (Alex, white, lecturer, Southeast)

“*The institution did not create guidelines for home office, leaving it to local coordinators and public servers’ discretion. Some public servers ended up overloaded, working beyond their workload. The institution did not provide psychological support to servers who worked under pressure and did not respond to questions on this topic*”. (Jeferson, white, admin staff, Southeast)

The battlegrounds were more dispersed during the pandemic. The symbolic code of life and death was reiterated by the proliferation of neglect, oppression and violence that followed the pace of the COVID-19 pandemic in Brazil. The experiences shared by Alex and Jeferson show distinctive differences across HEIs, from departments to personal levels. Alex’s colleagues suspended classes under individual decisions as a practice authorised by the institution and mainly as practice showing the relationality to neoliberal policy prescriptions that confused, as presented in the previous sections. The choices to promote clear guidelines and social policy shaped the pandemic by shaping social inequality (Bambra; et al., 2021, p. 86). Jeferson pointed to the consequences of such choices, as the colonial occupation established the borders of oppression in everybody of those working and studying at Brazilian public HEIs. Responding with “no”, adjusting or imposing limits, as suggested by Maísa, became a literal practice of warfare.

*The pandemic is costing human resources, not just about the number of deaths, but people are going to great lengths to meet demands that have arisen in the pandemic along with demands that already existed before*. (Gabriela, Black, student, South)

*Feelings of inability and inadequacy to the new format of classes messed with my mental health.* (Ursula, Black, student, Northeast)

*22% of the permanent staff (administrative and teaching) of the institution contracted COVID in loco. Two loved ones passed away. Maintaining face-to-face administrative work during the pandemic was a shame. Also, they cut 50% of the positions (administrative and temporary professors), which caused anxiety and frustration in the midst of the pandemic.* (Tamiris, white, lecturer, Southeast)

*The pandemic and its management in Brazil was a disaster. It greatly intensified all the inequalities already present on the national scene. Particularly, in a work that I developed with the prison population in the country’s northern region (Altamira-PA), I saw new forms of racism that strategically used the pandemic to carry out the Black genocide within the prison regime. My work was interrupted because of the pandemic.* (Paulo, Black, lecturer, Northeast)

The participants pointed to the efforts to continue with the HEIs activities and the emotional laden that it represented. At the same time, their lives were being affected by the COVID-19 as they contracted the virus, had to carry responsibilities to family members who contracted the virus or were grieving for those who did not survive the disease. The small-scale responses to the pandemic were dealing with the effects of COVID-19 and the consequences of the necropolitical federal administration. As Sylvia Wynter (2003) points out, the microlevel of the individual or the macro level of society functions within the regime of truth defined by the coloniality of being/power/truth/freedom, as she paraphrases Quijano (2000).

Adopting limitations, saying no and promoting self-care were observations made by the participants pointing to the empathy that occurred during the pandemic:

*The solidarity, acceptance, and care I received from my teachers. Both my supervisor and the teachers of the subjects I took.* (Rebeca, white, student, Southeast)

*Although I do not say that all relationships are easy, I received support from colleagues, management and students while I was facing COVID and supporting my sick parents*. (Sofia, white, lecturer, Southeast)

The students showed articulated narratives with political solidarity, pointing to organised networks to put pressure on funding organs and the HEIs and an acknowledgement to the staff and scholars working to provide and maintain quality education. On the other hand, lecturers pointed to a smaller niche of people providing support to each other. There was no reference to the union or other organised institutions representing the collective interest as staff and scholars working in the public sector. This contrast shows the relation of race and class that situates the profile of HEIs public servants in Brazil in the Western and westernised white middle class space. Students and staff representative entities (unions and social movements) focussed protests in response to the research and fellowship cuts and mass layoffs during the pandemic.

Resisting to the necropolitical system was a consistent remark that participants expressed as an attempt to demonstrate the efforts in learning new methods and techniques of online teaching–learning without receiving appropriate training; building small, supportive system with colleagues who are empathetic and considerate/ committed and dedicated; and pushing for internal decisions at the department level to offer support to students in situation of social vulnerability. Yet, the efforts could not change the structural format of exclusions or decode the symbolic life and death (Wynter, 2003) that caused discrimination and inequalities within the public HEIs context. As Zenaide (white, student, North) pointed, it is not enough to have situated actions:

“*I believe that, in the context in which we live, social responsibility and adaptation must come before personal opinions*”.

What Bolsonaro called a “hysteria” to label the urgent policies and procedures to be put in place during the COVID-19 pandemic belittled the consequences that reinforced racism, classism, ableism, sexism and geographic exclusion in the public Brazilian Higher Education context. The confluence of these intersectional oppressions determines the necropolitics (Mbembe, 2003) in Bolsonaro’s administration that is manufacturing inequalities and divisive possibilities to tackle such a destructive colonial strategy. In general terms, the research, vaccine development (CoronaVac), vaccination procedures, publications and data produced about the COVID-19 in Brazil (Daniel Jones, 2021; Mello, 2020) were consistently dismissed by the president. The president dismissed the relevance of HEIs as organs producing solutions for the pandemic and continuous free quality education. Such lack of acknowledgement created divisions in dealing with the critical situation and challenges for organised strategies to dismantle the current necropolitical administration.

## Conclusions

The effects of COVID-19 on the Brazilian public HEIs were undeniably damaging to the structure of free education and quality research of impact. Bolsonaro consistently addressed the nation with exclusionary mechanisms under a colonial code of life and death that marks nonwhites in the space of Otherness (Wynter, 2003). The misleading discourses and privation of support disfranchised HEIs in taking official actions and supporting those in the most vulnerable situations. The use of the words “genocide”, “chaos”, “terrible” and “awful” by the participants to describe the current administration was not only addressed to the external context of the HEIs but how it affected the internal practices and limited the decisions to adopt effective counter-narratives.

The effect of exclusions in public HEIs mirrored in contextualised proportions the racist, classist, ableist, sexist, transphobic and geographically exclusionary model present in the national reality. Even with the attempt to create support and care for staff, scholars and students, HEIs suffered the backlash of the irresponsible government at the same time that reproduced the colonial legacy that postulates the same exclusion system they are trying to tackle. The cyclic exclusion system compartmentalised solidarity, care and support that did not reach the institutional level and promote structural change. The potential way to strengthen the collective voices could be the more consistent presence of organised entities such as unions and social movements to address the experienced issues. Technological exclusion added to the social vulnerability demobilises these political articulations, having isolated moments that voiced the students’ struggle and the mass layoffs in HEIs during the pandemic.

Finally, the recommendations from this paper aim to push the conversation further towards institutional accountability at federal and state levels, community participation in the decision-making process and strategy to tackle inequalities taking an intersectional framework into account. I acknowledge that this paper has limitations concerning the small sample that did not intend to offer generalisations and comparisons of different regions in Brazil. However, it is not to confine the discussion to this perspective but offer support to further research in necropolitical governmental discourses during the pandemic. Also, more studies to understand alternative pedagogical methodologies developed by the Brazilian public HEIs taking into consideration regional disparities of public funds distribution and autonomy will be relevant for the changing scenario in teaching–learning experiences required during the pandemic. More comprehensive samples can help understand how scholars, students and staff are experiencing the development of hybrid teaching–learning methods in the following years.

While a central feature of this paper has been to illuminate the experiences of scholars, staff and students of Brazilian public HEIs, there is an onus on HEIs to develop more straightforward guidelines about the commitment to social equality, using their administrative autonomy. A precise political engagement means to stand in the antiracist struggle organised by PoC, in the struggle of people with disability, solo mothers, intersex and trans people, and beyond the groups cited in this work who are part of dissident identities. Joining their struggles for access, inclusion, visibility, representation and acceptability (Kuppan, 2018) shakes the boundaries of the colonial occupation within the HEIs. The engagement must question the design of practices that reinforce the code of life and death (Wynter, 2003) in HEIs, as a refusal to continue reproducing normalised administrative procedures that further excludes marginalised groups.

What must be contested is the consequence of exclusionary practices in Brazilian public universities. Participants disapproved of the current federal administration, empirically proving that racial disadvantages intersect with gendered, geographical, (dis)abled praxis of minoritisation. The information gathered in this research points to new requirements for institutional change. Implementing a constant monitoring mechanism for students, scholars and staff to improve methods of data records and safety for trans-inclusive procedures could be a transformative change of institutional practices. Regarding university spaces, it is crucial to offer alternative times to access the library and study rooms for students to have digital access. From this example, the universities would also guarantee the employment of professionals who lost their jobs during the pandemic at the public HEIs. Keeping library spaces open also enables the new pedagogical format to be adopted as new methodological praxis of hybrid learning. The central concern is how historically marginalised students can access, continue and finish their programmes at Brazilian public HEIs.

From a different perspective about the use of public HEIs spaces, the university refectories are important elements to safeguard the permanency of students in the university, especially those living in vulnerable financial situations. Not only, but especially during the pandemic, the refectories can be accessed as food banks for vulnerable students and the surrounding community. Food security in HEIs is another topic for further research, considering the disparity between Brazil’s new world record in food production (CNN, 2022) compared to the highest food insecurity globally (Exame, 2021). Poor food distribution affects educational access across all levels, including the HEIs (BBC, 2021; Maciel et al., 2022; UFMG, 2021). Aligned with the participants’ narratives, the lack of food access intersects with unemployment rates affecting students’ continuity in the programmes. Enabling food access is part of the accountability that public universities should have as a space for the encounter of knowledge and life.

The intention is not to offer solutions but venues towards a more accountable and just educational system that is free, yet not for all. The public administration in Brazil, from the local institutions such as HEIs to the federal government, needs to consider the knowledge produced “in the house” by researchers and research centres, activists and non-governmental organisations to assess the current issues that obstruct the promotion of social justice. The efficient use of data produced about the racial, social and economic conditions imposed during the pandemic is crucial to establishing new parameters to support the new social policy designed during and beyond the pandemic.

The neoliberal discourse of austerity to justify shutting down social programmes, such as affirmative actions and research funds, blocks the validation of lives, minoritised by a necropolitical administration. Negligent praxis also obstructs the expansion of knowledge production from more than those only framed by and through coloniality of power (Quijano, 2000). Disrupting the necropolitical agenda with a framework grounded in social justice promotion can create tolls to crack the colonial structure of power. It removes destructive barriers to access of delivering education at the institutional level. Along with the institutional support from HEIs, having organised organs to amplify the struggle of scholars, staff and students can be the key to operationalising transformation mechanisms. Strategising practices of dissent “onto the possibility of our fully realised autonomy of feelings, thoughts, behaviors” (Wynter, 2003, p. 331) is key to helping dismantle the colonial occupation.

## Footnotes

1 The census is developed by the Brazilian Statistics Institute (IBGE) every 10 years. The 2020 census was postponed due to a cut on the funds and the pandemic. The Ministry of Economy released an inform saying that the suspension could not predict a new date for data collection. In 2021, Bolsonaro vetoed a new census and sanctioned new cuts for the funds passed to IBGE (Globo G1, 2021a). This affects the new design of social policies and the lack of precise information about the population clouds, among other pieces of data, the mortality rate—the ultimate nature of this government’s agenda: necropolitics.
